# The Complex Interaction of Matrix Metalloproteinases in the Migration of Cancer Cells through Breast Tissue Stroma

**DOI:** 10.1155/2014/839094

**Published:** 2014-03-27

**Authors:** Kerry J. Davies

**Affiliations:** Canniesburn Plastic Surgery Unit, Glasgow Royal Infirmary, Castle Street, Glasgow G4 0SF, UK

## Abstract

Breast cancer mortality is directly linked to metastatic spread. The metastatic cell must exhibit a complex phenotype that includes the capacity to escape from the primary tumour mass, invade the surrounding normal tissue, and penetrate into the circulation before proliferating in the parenchyma of distant organs to produce a metastasis. In the normal breast, cellular structures change cyclically in response to ovarian hormones leading to regulated cell proliferation and apoptosis. Matrix metalloproteinases (MMPs) are a family of zinc dependent endopeptidases. Their primary function is degradation of proteins in the extracellular matrix to allow ductal progression through the basement membrane. A complex balance between matrix metalloproteinases and their inhibitors regulate these changes. These proteinases interact with cytokines, growth factors, and tumour necrosis factors to stimulate branching morphologies in normal breast tissues. In breast cancer this process is disrupted facilitating tumour progression and metastasis and inhibiting apoptosis increasing the life of the metastatic cells. This paper highlights the role of matrix metalloproteinases in cell progression through the breast stroma and reviews the complex relationships between the different proteinases and their inhibitors in relation to breast cancer cells as they metastasise.

## 1. Introduction

Cancer mortality most often results directly from metastatic spread to distant organs [1–3]. Approximately 6–10% of patients will present with metastatic disease at diagnosis. The median survival rate for these women is two to four years [4]. Historical records of women with inoperable breast cancers in London between 1805 and 1933 showed that 44%, 18%, and 4% of women were alive at three, five, and ten years, respectively. The median survival time was 2.7 years [4]. Today, the mean survival rate for highly metastatic breast cancers has not significantly changed with modern therapy being only effective in palliating symptoms and enhancing the quality of life [5].

Breast carcinomas spread in several ways. They can move directly into the skin and muscle, via lymphatics and other lymph nodes and via the blood stream to lungs, bone, liver, and brain [6]. The presence of metastasis in the axillary lymph nodes predicts the development of distant metastases; however, 20–30% of patients who are axillary node negative go on to develop metastases at other locations indicating that tumour cells bypass the lymph nodes and can disseminate directly through the blood to distant organs [2].

Whatever the route of metastasis, the tumour cells must first migrate through the local tissues. Noninvasive breast cancers remain within the basement membrane of the terminal duct lobule. Invasive breast cancers involve the dissemination of cancer cells outside the basement membrane of the duct and the lobules into the surrounding adjacent normal breast tissue stroma [7]. Changes in the malignant cells are often accompanied by alterations in the supporting myoepithelium and stroma cells due to a combination of events leading up to the invasion of the stroma, angiogenesis, and eventual breakthrough into the lymphatics or blood vessels [8]. This review aims to highlight some of the main processes involved in cell movement through the local breast tissue stroma.

## 2. Discussion

### 2.1. The Extracellular Matrix

The extracellular matrix (ECM) serves as a medium for cell-cell interactions and can directly signal cells through cell surface ECM receptors such as integrins. In addition, many growth factors and signalling molecules are stored in the ECM [9]. Cell-cell and cell-extracellular matrix interactions are essential in the development and maintenance of normal tissue cytoarchitecture and play an important role in the development and progression of many types of cancer [10]. Tumour cell metastasis represents an extremely complex process consisting of multiple sequential steps. The metastatic cell must exhibit a complex phenotype that includes the capability to escape from the primary tumour mass, invade the surrounding normal tissue, and penetrate into the circulation [11]. Tumours can be defined by their uncontrolled and invasive growth, but their phenotype is regulated in a complex fashion based on interactions of the malignant cells within the tumour stroma including the ECM, the vasculature, and the resident immune system [12]. Initially a cell within a colony is instructed to disrupt cadherin-based intercellular junctions and acquire a fibroblastoid, motile phenotype, initiating detachment from the primary site. This is enhanced by proteases which digest the basal lamina components and facilitate cell movement through the ECM [13].

### 2.2. Matrix Metalloproteinases

The matrix metalloproteinases (MMPs) are a family of zinc dependent endopeptidases [14]. To date there are at least 28 MMPs identified with fourteen implicated in breast cancer development and progression [15]. They are synthesised both by tumour and peritumoural stromal cells [16] and their primary function is degradation of proteins in the ECM [17] and they can be considered the most important proteolytic enzyme for connective tissue dissolution. MMPs are implicated in cancer invasion and metastasis with different classes of MMPs being associated more frequently with cancers of varying origin [18]. The major source of MMP activity in a tumour is from the surrounding stromal cells and it is likely that cancer cells are able to stimulate production by fibroblasts in a paracrine fashion [11].

In the normal breast, cellular structures change cyclically in response to ovarian hormones. Cell proliferation, apoptosis, invasion, and differentiation are integral processes that are precisely regulated but become dysregulated in pathologies such as cancer. ECM remodelling is a prerequisite to ductal progression and MMP activity facilitates this progression by removing or breeching the basement membrane and stromal matrix [19]. MMPs can be divided into four groups based on substrate specificity and domain organisation: the interstitial collagenases, the gelatinases, the stromelysins, and the membrane type MMPs [16].

### 2.3. Interstitial Collagenases

These MMPs, including MMPs 1, 5, and 13, catalyse the degradation of fibrillar forms of collagen, types I, II, and III [16]. Elevated levels of MMP 1 have been shown to be correlated with the depth of tumour invasion, angiogenesis, lymphangiogenesis, and the presence of local and distant metastases in breast cancer [20]. Elevated expression of MMP 1 can promote the local growth and the formation of brain metastases by breast cancer cells [21]. MMP 1 has been shown to have an independent prognostic value in breast cancer and both tumoural and stromal expression are associated with breast tumour progression and poor prognosis [22].

Collagenase-3 (MMP 13) degrades helices of fibrillar collagens with preferential activity on type II collagen [23]. It is induced by many factors including IL-1 and transforming growth factor *β* (TGF-*β*) and is activated through a proteolytic cascade involving other MMPs. It exhibits a broad spectrum of activity against the ECM. It is released from stromal fibroblasts and facilitates tumour growth through regulating the activity or availability of growth factors sequestered as inactive molecules in the ECM. In breast cancer it is implicated in an uncontrolled degradative process occurring during tumour progression [23]. Within the inflammatory bone microenvironment MMP 13 production was upregulated in breast tumour cells leading to increased preosteoclast differentiation and their subsequent activation [24].

Attachment to laminin is a key event in the process of local and vascular invasion. In order for vascular invasion to occur, laminin receptors have to be upregulated. They are normally upregulated by cytokines, inflammatory agents, and ECM proteins such as fibronectin [25]. Fibronectin is found throughout the body so it is highly regulated by various integrins, a family of various *α*/*β* fibronectin receptors such as the *α*_4_ *β*_1_ surface receptor that mediates cell-matrix and cell-cell interactions by attachment to laminin. MMP 1 was found to upregulate *α*_5_ *β*_1_ receptors which lead to a reduced number of *α*_4_ *β*_1_ fibronectin receptors relative to mammary epithelial cells [20]. MMP 1 cleaves collagen molecules into fragments that are unstable at body temperature and unwind and denature into constituent collagen *α* chains. The denatured *α* chains are susceptible to hydrolysis by the gelatinases (MMPs 2 and 9) [14]. This can lead to aggressive cancers with increased incidence of invasion and of metastasis [20].

### 2.4. Gelatinases

The gelatinases, for example, MMPs 2 and 9, are type IV collagenases that degrade gelatine (denatured collagen) and types IV, V, VII, IX, and X collagen. Type IV collagen is particularly abundant in basement membranes [16]. In a study using mammary tumour bearing mice there was a dramatic upregulation of MMP 9 secretion by splenic and tumour infiltrating T-lymphocytes suggesting that tumour cells may use inflammatory cells to make contributions to the tumour phenotype [26]. MMP 9 has been shown to exert both pro- and antitumourigenic properties. MMP 9 activates tumour infiltrating macrophages into a tumour inhibiting phenotype [27]. Elevated serum levels have been found to be associated with tumours and correlate with cancer invasion and metastasis [12]. In breast cancer, TGF-*β* signalling was shown to decrease growth of the primary tumour but promote metastasis. Inhibition of TGF-*β* signalling has been shown to decrease metastasis of mammary tumours by impairing invasion, migration, and cellular survival and may prove to be a potential antimetastatic therapy in the future [28].

A complex variety of cytokines and growth factors such as TGF-*β*, hepatocyte growth factor (HGF), epidermal growth factor (EGF), and tumour necrosis factor *α* (TNF*α*) have been shown to influence MMP 9 induction by fibroblasts in breast cancer cells [12]. Cell contact between breast cancer cells results in the rapid release of inactive membrane associated MMP 2. MMP 2 is abundantly expressed at tumour leading edges in breast cancer and contributes to cell migration across collagen type I. Once released MMP 2 may then associate with other MMP complexes facilitating its activation and subsequent invasion of normal tissues by malignant cells [29].

### 2.5. Stromelysins

Stromelysins, for example, MMPs 3, 7, 10, and 11, have broad substrate specificity, catalysing degradation of many different substances. These include proteoglycans, noncollagenous proteins such as laminin and fibronectin [16]. MMP 3 is produced by senescent fibroblasts induced by HGF and stimulates epithelial cell proliferation and is critical for branching morphologies, especially secondary and tertiary branching of the lobules in the differentiating mammary gland [30].

### 2.6. Membrane Type MMPs

This group of MMPs possesses a membrane-spanning domain and has been shown to catalyse the activation of progelatinase-A which degrades a variety of ECM substances and functions as a fibrinolytic enzyme [16]. The most common MMP in this group, membrane type 1 MMP (MT1-MMP), interferes with the hosts' immune system by inactivating C3b and C4b and removing them from the cell surface of tumour cells [31]. This inhibits the activation of the complement system against the tumour cells by preventing recognition by phagocytic and natural killer cells. In addition they deactivate C3a which would normally potentiate antitumour responses via their chemoattractant and proinflammatory activity. MT1-MMP is also an activator of MMPs 2, 13, and 14. The proteolytic activity is directly related to cell migration [32] so expression of MT1-MMP is associated with aggressive, invasive malignant cells.

### 2.7. Regulation of MMPs

Extracellular matrix metalloproteinase inducer (EMMPRIN) is one of the molecules involved in the regulation of several MMPs including MMPs 1, 2, and 3 [25] and MT1-MMP in fibroblasts [11]. In patients with breast cancer with metastases to the pleural space EMMPRIN was found to be upregulated leading to increased expression of several MMPs, with MMP 2 being most affected [25]. EMMPRIN has been shown to colocalise with the *α*_3_ *β*_1_ and *α*_6_ *β*_1_ integrins at the cell membrane leading to attachment to laminin and subsequent local invasion of tissues [25].

Tissue inhibitors of metalloproteinases (TIMPs) inhibit protease activity by forming high affinity noncovalent complexes with active MMPs. Four inhibitors have been described; TIMPs 1, 2, 3, and 4 [16]. TIMPs are abundant in mammary tissues as they are required for the structural and functional changes that occur during the menstrual cycle and pregnancy [19]. In breast cancer, epithelial cell proliferation and ECM remodelling are dysregulated facilitating tumour progression and metastasis [19]. TIMP 1 controls mammary epithelium proliferation through limitation of matrix degeneration. TIMPs 1, 3, and 4 are localised to mammary stromal cells and surrounding ducts and TIMP 4 is also present in breast adipocytes [19]. TIMPs 1 and 2 have been shown to inhibit apoptosis [16]. Overexpression of TIMP 1 inhibits apoptosis after the loss of cell adhesion in human breast cancer cells [33] increasing the cell life of motile metastatic cells. These findings show that TIMPs may not be appropriate targets for future drug therapy [16] as the most important determinant is the precise balance between the production and activation of proteinases and the production of their inhibitors in the metastatic potential of breast cancers [34].

### 2.8. Macrophages

High levels of macrophage infiltrate in breast tumours correlate with a poor prognosis and hypoxic tumours have a higher invasive capacity and poorer prognosis than well-oxygenated tumours [35]. Macrophages secrete MMP 9 and their podosomes are capable of directly degrading the pericellular ECM [36]. Macrophages also promote tumour invasion by secreting uPA (urokinase plasminogen activator). uPA initiates a proteolytic cascade that results in the conversion of plasminogen to plasmin which mediates proteolysis [37]. uPA further recruits MMPs 9 and 2 enabling the remodelling of collagen type IV, the major constituent of basement membranes [38]. Breast tumour cells are then free to flow out of the ductally constrained tumour mass into the surrounding stroma and subsequently gaining access to the vasculature with the ability to colonise distant sites. High levels of uPA are associated with poor prognosis in breast cancer and an elevated serum uPA is an established prognostic factor used for determining treatment-based decisions in early breast cancer [37].

### 2.9. Epithelial Mesenchymal Progression

Epithelial-mesenchymal transition (EMT) is a development process in which epithelial cells take on the characteristics of invasive mesenchymal cells [38]. Evidence has demonstrated an important role of EMT pathways in the progression of carcinoma to metastasis providing the epithelial tumour cells with the ability to migrate, invade the stroma, and disseminate [39]. EMT like changes correlate with a more aggressive phenotype [40]. Elevated levels of MMPs in the tumour microenvironment can directly induce EMT in epithelial cells. Cancer cells undergoing EMT can then produce more MMPs facilitating cell invasion and EMT can generate activated stromal like cells which drive cancer progression via further MMP production [38].

The Breast Cancer Health Disparities study evaluated genetic variation in MMPs 1, 2, 3, and 9 and breast cancer risk. MMP 1 and MMP 2 were associated with breast cancer overall and were associated with oestrogen receptor positive/progesterone positive cancers and with oestrogen positive/progesterone receptor negative tumours. MMP 3 and MMP 9 were associated with oestrogen and progesterone receptor negative tumours. These findings suggest that genetic variation in MMP genes influences breast cancer development and survival [40].

## 3. Conclusion

The aim of current research is to identify future treatments for breast cancers by understanding the mechanisms involved in metastatic spread in order to identify potential drug therapies. Unfortunately, to date, research has had to consider specific cancer cell lines in isolation. Breast cancer is not one specific entity, but, by the very nature of the cellular changes, neoplasms are complex and multifactorial with neoplasms displaying characteristic genetic instability with divergent properties even within a single tumour. This will mean resistance to single focus drug therapy. Identifying a rate-limiting step in the complex mechanism to target drug therapy has not yet been possible mainly due to the cellular toxicity of synthetic MMP inhibitors caused by complex interactions with other tissues throughout the body. MMPs may also prove to be useful for prognostic and predictive markers of metastatic disease, enabling doctors to identify high-risk cancers and tailoring therapy accordingly.

Future research may one day locate the rate limiting steps that breast cancer cells need to adopt a malignant phenotype. Targeting these mechanisms, so that healthy tissues are not disrupted, could mean that tumour cells would remain constrained within the local tissues. As tumour invasion is the most important diagnostic criteria for malignancies, then by definition the tumour would no longer be malignant. As breast cancer is the largest cause of deaths in women aged 35–55, this would be a ground breaking achievement that would significantly reduce mortality and morbidity associated with breast cancer.
